# Does the Serratus Plane Block Added to the Interscalene Block Improve the Quality of Anesthesia in Arthroscopic Shoulder Surgery? A Prospective Randomized Study

**DOI:** 10.7759/cureus.7648

**Published:** 2020-04-12

**Authors:** Ufuk Demir, Ahmet Murat Yayik, Mehmet Köse, Muhammed E Aydin, İrem Ates, Ali Ahiskalioglu

**Affiliations:** 1 Anesthesiology and Reanimation, Kastamonu State Hospital, Kastamonu, TUR; 2 Anesthesiology and Reanimation, Ataturk University School of Medicine, Erzurum, TUR; 3 Clinical Research, Development and Design Application and Research Center, Ataturk University School of Medicine, Erzurum, TUR; 4 Orthopaedics and Traumatology, Ataturk University School of Medicine, Erzurum, TUR; 5 Clinical Research, Development and Design Application and Research Center, Ataturk University, Erzurum, TUR

**Keywords:** serratus plane block, interscalene brachial plexus block, arthroscopic shoulder surgery, ultrasound-guided

## Abstract

Background: Interscalene brachial plexus block (ISBPB) is the gold standard method in shoulder surgery. Serratus plane block (SPB) provides anesthesia in hemithorax, axillary region, and posterior of the shoulder. This randomized controlled study evaluated the effect of SPB added to ISBPB on surgical anesthesia quality in arthroscopic shoulder surgery.

Methods: Sixty patients undergoing arthroscopic shoulder surgery were randomly assigned to two groups. All surgeries were performed under regional anesthesia. The Group I (Group Interscalene) (n=30) received ultrasound-guided interscalene block. In the Group IS (Group Interscalene + Serratus) (n=30), ultrasound-guided interscalene block and SPB were performed. Intraoperative anesthetic agent consumption, postoperative opioid consumption, postoperative pain scores, patient satisfaction, and surgeon satisfaction were evaluated.

Results: Intraoperative propofol (60.00 ± 45.49 vs. 24.00 ± 32.97, respectively) and fentanyl (33.33 ± 23.97 vs. 18.33 ± 24.51, respectively) consumption were significantly higher in Group I than in Group IS (p < 0.05). There was no statistically significant difference between the groups at any of the times the postoperative opioid consumption and pain scores were evaluated (p > 0.05).

Conclusions: SPB added to the ISBPB increases the quality of surgical anesthesia and reduces the need for intraoperative sedoanalgesia for arthroscopic shoulder surgery.

## Introduction

Arthroscopic shoulder surgery, which has been applied frequently in recent years, provides long-term positive clinical results and permanent pain relief but can cause severe pain in the early postoperative period [1-2]. Postoperative pain management is critical in shoulder arthroplasty. It promotes rapid rehabilitation and can contribute to a successful surgical outcome [1].

Even though it is possible to rely on opioids in the perioperative analgesia for shoulder arthroplasty, their side effects are alarming. Therefore, regional anesthetic methods are frequently preferred as part of multimodal analgesia to provide better pain control and to reduce the side effects associated with opioids [3]. In shoulder arthroplasty, isolated nerve blocks such as suprascapular and axillary nerve blocks for postoperative analgesia are frequently applied as well as the brachial plexus blocks from the costoclavicular, supraclavicular, and interscalene regions [1, 4-5]. Interscalene brachial plexus block (ISBPB) is accepted as the gold standard in shoulder surgery. ISBPB provides excellent postoperative analgesia when given in combination with general anesthesia in shoulder arthroplasty. Furthermore, it can be used in surgical anesthesia together with sedation [6]. However, as the posterolateral branch of the shoulder also receives a sensory branch from the thoracic nerves, an arthroscopy port entry in this region in the arthroscopic surgery may cause pain to the patient.

Serratus plane block (SPB) was first defined in 2013 by Blanco et al. This block provides anesthesia and analgesia in the hemi-thorax, where it is applied to block the thoracic intercostal nerves, in addition to the axillary region and shoulder posteriorly [7]. In shoulder surgery, if the thoracic innervation is blocked inadequately, especially in the manipulations of the posterior side of the glenoid capsule, the patient may experience pain. SBP can also be used in shoulder surgery with adequate thoracic innervation blockade [8].

The aim of this study was to investigate the effect of ultrasound-guided SPB added to ISBPB in shoulder arthroplasty on intraoperative anesthetic agent consumption, postoperative opioid consumption, and pain scores.

## Materials and methods

After approval of the local ethics committee (Ethical Committee Ataturk University, Erzurum, Turkey), 60 patients aged between 18 and 65 years, American Society of Anesthesiologists (ASA) I-II scheduled for arthroscopic shoulder surgery were included the study.

Patients with known severe heart, kidney, liver, or hematologic diseases and peptic ulcer, gastrointestinal bleeding, central or peripheral neurologic disease, psychiatric disorders, drug allergy or a history of allergy to amide-type local anesthetics, a history of chronic pain, routine analgesic use, lower 45 kg in weight, contralateral phrenic nerve paralysis, or declining to take part in the study were excluded.

Microsoft Office 365 Excel (Microsoft, Redmond, WA, USA, http://www.microsoft.com) was used for the generation of the random allocation sequence to assign the subjects to two groups: the ISBPB group (Group I, Group Interscalene), the interscalene brachial plexus and SPB group (Group IS, Group Interscalene + Serratus). All patients were taken to the regional anesthesia room 20 min before the operation and vascular access was established by a 20 G intravenous catheter, crystalloid infusion was started at 6 mL/kg. Standard monitoring was performed with pulse oximetry, noninvasive arterial blood pressure, and electrocardiogram monitoring.

**Figure 1:**
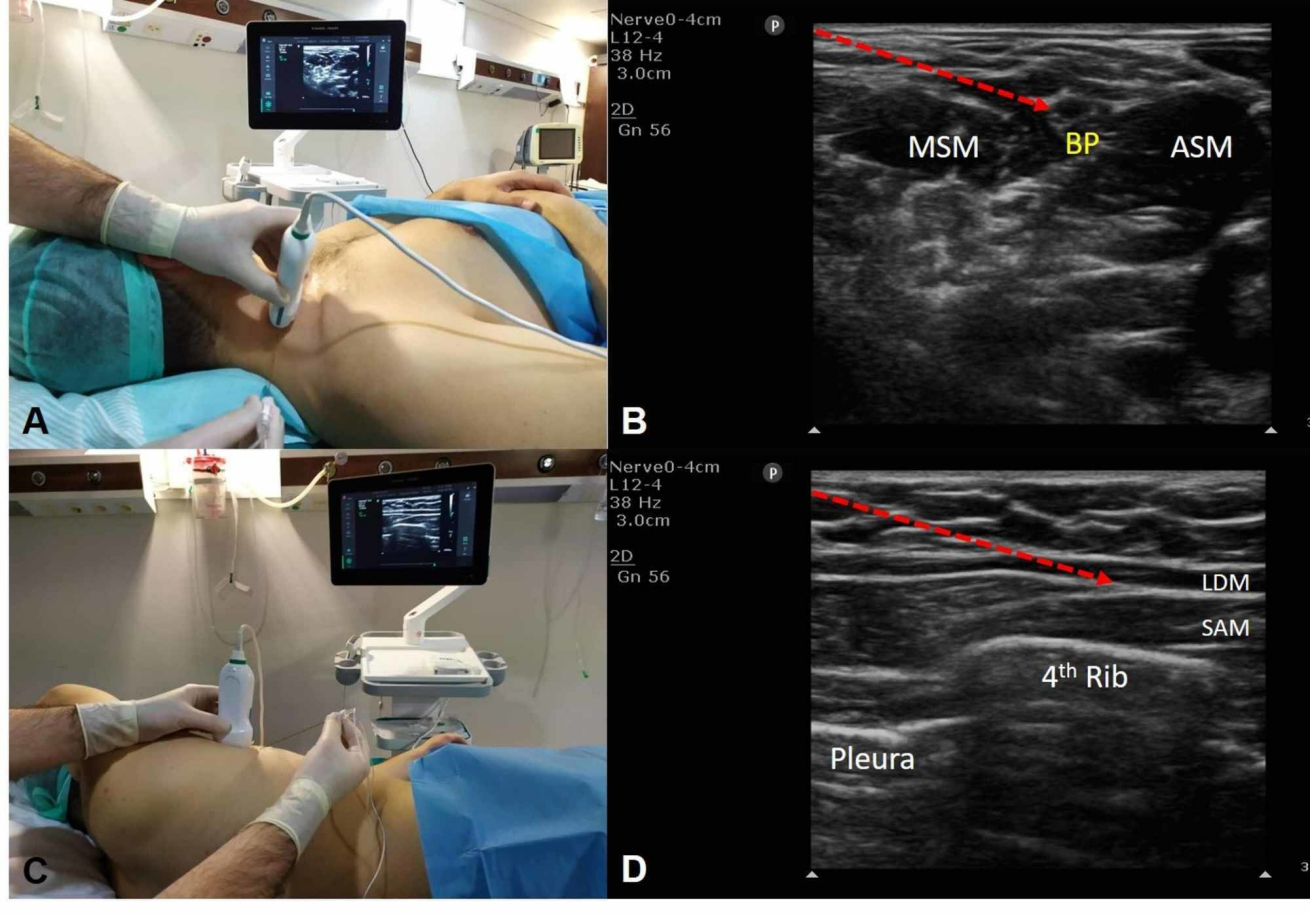
Ultrasound-guided block procedure. (A) Probe and ultrasound set up for ISBPB. (B) Sonographic anatomy of the block, MSM, ASM, and BP. (C) Probe and ultrasound set up for SPB. (D) Sonographic anatomy of the block, LDM and SAM. ISBPB, interscalene brachial plexus block; MSM, middle scalene muscle; ASM, anterior scalene muscle; BP, brachial plexus; SPB, serratus plane block; LDM, latissimus dorsi muscle; SAM, serratus anterior muscle.

In Group I (n: 30): patients received only ISBPB. The patient's head was turned to the opposite side while the patient was in the supine position and the shoulder to be treated was elevated 5-10 cm. The high-frequency linear ultrasound probe (12-4 MHz) and the region to be treated were prepared in sterile conditions and placed transversely across the external jugular vein at 3-4 cm above the clavicle (Figure 1A). Between the anterior and middle scalene muscles, the imaging of the brachial plexus showed three to five hypoechoic circles. The entry was with the in-plane technique using an 80-mm block needle from lateral to medial. After confirming the needle insertion site with 2 mL saline solution, a 20 mL block fluid (2% lidocaine at 10 mL, 0.5% bupivacaine at 10 mL, and 1/200,000 adrenaline) was administered (Figure 1B).

In Group IS (n: 30): patients received an ISBPB and SPB. ISBPB was applied with the same technique as described in Group I and 20 mL (2% lidocaine at 10 mL, 0.5% bupivacaine at 10 mL, and 1/200,000 adrenaline). After the interscalene block, the patient was placed in the lateral decubitus position with the area to be treated on the upper side. The high-frequency linear ultrasound probe and the region to be treated were sterilized. The ultrasound probe was placed on the anterior axillary line at the level of fourth and fifth ribs (Figure 1C). Images of the muscles latissimus dorsi and serratus anterior, the ribs, and the pleura were obtained. Subsequently, with the in-plane technique, an 80-mm block needle was advanced between the latissimus dorsi and the serratus muscles planes in a caudal to the cranial direction. There was no blood or air in aspiration. After confirming the location of the needle with 2 mL of saline solution, a mixed solution of 30 mL (2% lidocaine at 15 mL, 0.5% bupivacaine at 15 mL, and 1/200,000 adrenaline) was administered between the two muscles (Figure 1D).

In order to avoid local anesthetic toxicity, lidocaine was limited to 7 mg/kg, bupivacaine to 2 mg/kg, and the fluid was topped with saline solution to obtain the desired volume. An examination was performed 20 min after the block to confirm the success of the block with total inability to move the shoulder.

The patient was taken to the operation room, placed in beach-chair position, and 2-4 L/min O2 was started. For arthroscopic surgery, all patients underwent a standard of three arthroscopic port entries in the anterior, lateral, and posterior positions. The patients who felt pain during the surgical incision, those with a 20% increase in heart rate and arterial blood pressure, or a 15% increase in the respiratory rate received 50 mcg fentanyl. For those patients who could not tolerate surgery after the administration of fentanyl, an infusion of 1-2 mg/kg propofol per hour was started and recorded. Patients who could not tolerate surgery even after propofol and fentanyl were given a single dose of 50 mg ketamine, and where they still could not tolerate it, a general anesthesia protocol was initiated.

Postoperative analgesia

The same protocol was applied in both groups for postoperative analgesia. Both were given 50 mg dexketoprofen trometamol before the surgery was completed. Postoperatively, this was repeated every 12 h. Patients were transferred to the post-anesthesia care unit (PACU) after the surgery and connected to a patient-controlled analgesia (PCA) device. The PCA device prepared with fentanyl was programmed at a concentration of 10 mcq/mL, without a loading dose and basal infusion, and with a lock-up time of 15 min, 25 mcq demand. Patients with an Aldrette score of 9 and above were transferred to the ward. Postoperative opioid consumption was evaluated as 0-4, 4-8, 8-24, and 24 h in total.

Postoperative pain evaluation was performed at the PACU using the Visual Analog Pain Scores (VAS) in hours 2, 4, 8, 12, and 24. Patients with a VAS score of four or above received 25 mg intravenous meperidine as a rescue analgesia.

 Patient satisfaction was evaluated at the end of the hour 24 according to the following four-point scale: 1. Excellent (no pain or discomfort), 2. Good (mild pain or discomfort, but no need for analgesia), 3. Moderate (pain tolerated with additional analgesic), 4. Poor (severe pain that cannot be tolerated even with analgesic). As for the intraoperative satisfaction of the surgeon, it was evaluated at the end of the surgery on a three-point scale: 1. Good, 2. Moderate, and 3. Poor.

Sample size determination

The sample size calculation was based on our ‎primary variable intraoperative propofol consumption. A preliminary study in our clinic and unpublished data indicated that propofol consumption should be 64.00 ± 40.88 mg in Group I, n = 10 and 24.00 ± 35.02 mg in Group IS, n = 10. A total sample size of 25 was calculated using G*Power version 3.1.9.2 with an alpha probability of 0.05 and a power of 0.95, and a large effect size (1.05) for the intraoperative propofol consumption. Considering possible dropouts, and for higher power, we decided to include at least 30 patients in each group.

Statistical analyses

Statistical analysis was performed using SPSS software Version 21.0 (IBM Corp., Armonk, NY, USA). The distribution of variables was evaluated for normality using the Kolmogorov-Smirnov test. Descriptive data were expressed as mean ± standard deviation. Normally distributed data comprising continuous variables were analyzed using the Student’s *t* test. Categorical variables were analyzed using the Chi-square test. p < 0.05 was considered statistically significant.

## Results

There was no statistically significant difference between the groups in terms of demographic characteristics, duration of surgery (p = 0.815), and duration of anesthesia (p = 0.398) (Table 1).

**Table 1:** Demographic and operative characteristics. Values are presented as number or mean ± standard deviation. I: Group Interscalene, IS: Group Interscalene + Serratus; ASA, American Society of Anesthesiologists. ^a^Independent sample *t*-test. ^b^Chi-square test.

	Group I (n=30)	Group IS (n=30)	p
Age (years)	44.67 ± 12.70	41.10 ± 11.67	0.262^a^
Weight (kg)	77.53 ± 11.42	78.33 ± 12.13	0.793^a^
Height (cm)	170.37 ± 9.31	169.70 ± 7.31	0,759^a^
Sex (F/M)	13/17	12/18	0,793^b^
ASA status (I/II)	23/7	20/10	0,390^b^
Duration of anesthesia (min)	97.50 ± 18.79	101.83 ± 20.61	0.398^a^
Duration of surgery (min)	68.83 ± 18.79	67.66 ± 19.64	0,815^a^

Intraoperative propofol (60.00 ± 45.49 vs. 24.00 ± 32.97, p = 0.001, respectively) and fentanyl (33.33 ± 23.97 vs. 18.33 ± 24.51, p = 0.020, respectively) consumption were significantly higher in Group I than in Group IS. There was no difference between the groups in their transition to general anesthesia (p = 0.492) and ketamine consumption (p = 0.671) (Table 2).

**Table 2:** Intraoperative anesthetic consumption. Values are presented as mean ± standard deviation. I: Group Interscalene, IS: Group Interscalene + Serratus. ^a^Independent sample *t*-test. ^b^Fisher's Exact Test.

	Group I (n=30)	Group IS (n=30)	p
Intraoperative fentanyl consumption (µg)	33.33 ± 23.97	18.33 ± 24.51	0.020^a^
Intraoperative propofol consumption (mg)	60.00 ± 45.49	24.00 ± 32.97	0.001^a^
Ketamine consumption (yes/no)	4/26	2/28	0.671^b^
Transition to general anesthesia (yes/no)	1/29	0/30	0.492^b^

Postoperative pain assessment was done at the PACU, 2nd, 4th, 8th, 12th, and 24th hours by using the VAS pain scale. There was no statistically significant difference between the groups at any of the times the pain score was evaluated (p > 0.05) (Table 3).

**Table 3:** Comparison of VAS at postoperative time points. Values are presented as mean ± standard deviation. I: Group Interscalene, IS: Group Interscalene + Serratus; PACU, post-anesthesia care unit; VAS, Visual Analog Pain Scores. ^a^Independent sample *t*-test.

	Group I (n=30)	Group IS (n=30)	p^a^
PACU	0.83 ± 1.60	0.43 ± 0.86	0.232
2 h	1.10 ± 1.71	0.63 ± 1.27	0.235
4 h	2.90 ± 1.67	2.13 ± 1.98	0.110
8 h	4.03 ± 1.71	3.40 ± 1.57	0.140
12 h	4.00 ± 1.78	3.70 ± 1.44	0.476
24 h	2.60 ± 1.48	2.07 ± 1.55	0.178

A comparison of the postoperative 24-h fentanyl consumption between Group I and Group IS also showed no significant difference within a range of 0-4 h (39.17 ± 48.99 vs. 23.33 ± 34.70, p = 0.154, respectively), 4-8 h (110.00 ± 67.47 vs. 95.00 ± 71.14, p = 0.405, respectively), 8-24 h (123.33 ± 74.26 vs. 111.67 ± 61.49, p = 0.510, respectively), and 24 hours’ total (272.50 ± 148.17 vs. 230.00 ± 134.45, p = 0.249, respectively) (Table 4). Surgeon satisfaction was evaluated at the end of the surgery, whereas patient satisfaction was evaluated at the end of the 24-h postoperative period. There was no statistically significant difference between the groups in terms of patient satisfaction (p = 0.415) and surgeon satisfaction (p = 0.132) (Table 4). 

**Table 4:** Postoperative fentanyl consumption, patient satisfaction, and surgeon satisfaction. Values are presented as mean ± standard deviation. I: Group Interscalene, IS: Group Interscalene + Serratus. ^a^Independent sample *t*-test. ^b^Chi-square test.

	Group I (n=30)	Group IS (n=30)	p
0-4 h (mcg)	39.17 ± 48.99	23.33 ± 34.70	0.154^a^
4-8 h (mcg)	110.00 ± 67.47	95.00 ± 71.14	0.405^a^
8-24 h (mcg)	123.33 ± 74.26	111.67 ± 61.49	0.510^a^
Total 24 h (mcg)	272.50 ± 148.17	230.00 ± 134.45	0.249^a^
Patient satisfaction (excellent/good/moderate/poor)	14/11/5/0	19/7/4/0	0.415^b^
Surgeon satisfaction (good/moderate/poor)	16/13/1	23/7/0	0.132^b^

During the administration of ISBPB and SPB or in the postoperative period, none of the patients suffered any complications including local anesthetic toxicity, vascular puncture, prolonged block, and nerve damage.

## Discussion

This study has shown that in the arthroscopic shoulder surgery anesthesia, the SPB added to the ISBPB improves the quality of surgical anesthesia and reduces the intraoperative consumption of sedoanalgesia agents. However, it does not affect postoperative pain scores and opioid consumption.

Shoulder arthroplasty may cause severe pain, especially in the first 24-h postoperative period. Postoperative pain control is crucial both for the patient’s comfort and for an effective rehabilitation in the early period after shoulder surgery. Shoulder arthroplasty can be performed with general anesthesia, regional anesthesia, or regional anesthesia added to general anesthesia. In postoperative analgesia strategies, the benefits of regional anesthetic methods exceed those of systemic opioids as they do not only provide more effective pain control, but also reduce opioid-associated side effects such as respiratory depression or nausea and vomiting [9]. ISBPB is the gold standard method for postoperative analgesia in shoulder surgery [10]. In patients who receive general anesthesia, ISBPB provides excellent postoperative analgesia, significantly reduces opioid consumption, and shortens hospital stay [10].

Beach-chair position is frequently used as it allows easier access to shoulder for the arthroplasty surgery. As a review of the field literature shows, it is well known that the beach-chair position for shoulder arthroplasty is associated with hypotension that can cause cerebral hypoperfusion [11-12]. Prolonged severe cerebral hypoperfusion can bring permanent neurological damage [13]. In patients under general anesthesia, advanced monitoring techniques such as near-infrared spectroscopy are required to evaluate cerebral perfusion [14]. Regional anesthesia has the benefit of making it possible to communicate with the patient during shoulder arthroplasty to keep informed about cerebral functions. Furthermore, in comparison to general anesthesia, regional anesthesia has the advantages of avoiding complications associated with endotracheal intubation and mechanical ventilation, decreased frequency of postoperative nausea and vomiting and early mobilization of the patient.

The ISBPB ensures the blocking of all motor nerves and most sensory nerves of the shoulder muscles providing excellent postoperative analgesia, which in turn can also provide surgical anesthesia combined with sedation [6]. Subsequently, the risks associated with general anesthesia can be avoided, especially in shoulder arthroplasty operations performed in the beach-chair position. In the shoulder arthroplasty, basically three arthroscopy port entries are performed: anterior, posterior, and lateral. The shoulder posterolateral inferior shows that this region also receives a sensory branch from the thoracic nerve [8]. ISBPB does not affect this region. As patients may experience severe pain during a posterior port entry, sedation may be required. In this type of surgery, performed in beach-chair position and in the proximity of the head, administration of deep sedoanalgesia also has its limitations.

The SPB is performed through a local anesthetic injection in the superior or inferior of the serratus muscle in the sagittal plane of the mid-axillary line at the level of the fourth and fifth ribs [7]. The injection is reported to ensure anesthesia in the hemithorax between the dermatomes T2 to T9 and in the axillary region and that it may be an alternative technique to central neuroaxial methods [7]. SBP is successfully administered for postoperative analgesia in breast [15] and thoracic surgery [16] and for surgical anesthesia in axillary mass [17] and thoracic wall mass [18] surgery. Furthermore, the literature includes case reports demonstrating its effectiveness against neuropathic pain [19]. When the dermatomal area affected by the SPB is examined, it is seen that it successfully secures anesthesia in the axillary region and postero-inferior of the shoulder, as well. For this reason, some authors argue that it can be used as a complementary block in shoulder surgery [8]. In our study, we showed that SPB added to ISBPB reduced the need for intraoperative sedoanalgesia by providing a better quality surgical anesthesia. However, when evaluated in terms of postoperative analgesia, there was no significant difference between the groups.

On the other hand, our study had some limitations. Firstly, propofol consumption was applied as standard in the sedation dose and bispectral index (BIS) monitoring was not employed to evaluate the depth of sedation and intra-operative anesthesia. Performing BIS monitoring and adjusting the depth of anesthesia accordingly could affect the results. Secondly, postoperative pain scores were evaluated in the first 24 h. Patients’ pain scores in postoperative rehabilitation and exercise might be higher and affect the results. Finally, the study was conducted as one blind only and Group I did not receive sham injection for the SPB and, therefore, the placebo effect of the injection could not be evaluated.

## Conclusions

This study has shown that in the arthroscopic shoulder surgery anesthesia, the SPB added to the ISBPB improves the quality of surgical anesthesia and reduces the consumption of intraoperative sedoanalgesia agents. In our opinion, with these benefits SPB may be a complementary technique preferred by most anesthetists for anesthesia management in arthroscopic shoulder surgery in addition to ISBPB.
